# Editorial for the Special Issue on Heat and Mass Transfer in Microchannels

**DOI:** 10.3390/mi17010126

**Published:** 2026-01-19

**Authors:** Xin Xiao, Xuan Zhang, Long Zhang

**Affiliations:** 1 College of Environmental Science and Engineering, Donghua University, Shanghai 201620, China; xin.xiao@dhu.edu.cn; 2 Department of Energy and Power Engineering, School of Mechanical Engineering, Beijing Institute of Technology, Beijing 100081, China

## 1. Introduction to the Special Issue on Heat and Mass Transfer in Microchannels

Research on heat and mass transfer in microchannels represents the cutting-edge frontier in modern high-density electronic cooling, advanced energy systems, and precision microfluidic manipulation [1,2,3]. By deeply integrating heat and mass transfer science with micro-/nano-fabrication technologies, it achieves highly efficient energy and material transport within extreme spatial constraints, underpinning a series of disruptive technologies that range from chip-level thermal management [4] to biosensing [5].

The strategic significance of this field lies not only in its irreplaceable compactness and efficiency, but also in its ability to reveal and harness unique physical mechanisms that dominate at the micro-scale, such as pronounced surface-tension effects [6], Marangoni convection [7], and capillary forces [8]. This lays the foundation for fundamentally innovating thermal management strategies and serves as a key driver for the continued miniaturization and performance leap of devices.

However, to fully unlock the potential of microchannel systems, a profound understanding and mastery of their inherent complexities is essential. This involves uncovering the dynamic transport laws at multiphase interfaces [9], analyzing the fine-tuning effect of complex geometries on flow and heat transfer [10], mastering the coupled multi-physics behavior during phase-change processes [11] and under extreme conditions [12], and promoting the innovative application of novel functional working fluids [13] and high-performance structures.

Therefore, the pursuit of efficient [14], stable, and controllable heat and mass transfer through microchannel technology has become a mission of both great scientific value and urgent engineering need. This requires that the design principles of microchannels be deeply coordinated with mesoscopic physical mechanisms, advanced materials [15], and fabrication processes. It is precisely this interdisciplinary integration that continuously pushes the field to break through bottlenecks and explore new technological frontiers.

**Figure 1:**
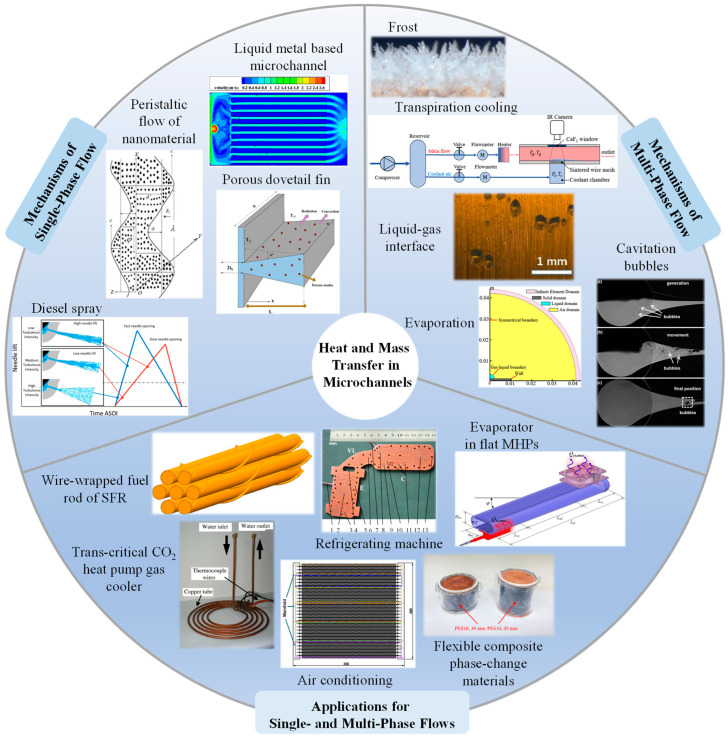
Topics covered in the Special Issue titled “Special Issue on Heat and Mass Transfer in Microchannels”.

The 17 original research articles included in this Special Issue can be clearly categorized into four main directions, based on their research subjects and orientations: fundamental studies on single-phase flow [Contributions 2, 5, 8, 15, 17], fundamental studies on multiphase flow [Contributions 1, 3, 4, 13, 14, 16], and applied research on both single-phase and multiphase flow [Contributions 6, 7, 9–12], as illustrated in Figure 1. This classification framework systematically presents the complete research spectrum from fundamental flow principles to engineering solutions.

In summary, the research presented in this Special Issue deepens the understanding of fundamental mechanisms in microscale flow and heat transfer and provides key insights and solutions for the design of next-generation microchannel heat sinks, micro-heat exchangers, and high-performance microfluidic systems. Future research will continue to advance toward intelligent design, cross-scale integration, and reliable applications in even more extreme environments.

## 2. Mechanisms of Single-Phase Flow

This group investigates the fundamental transport behavior in single-phase flows under complex conditions. Studies explore the influence of needle valve dynamics on near-nozzle spray characteristics [Contribution 2], analyze magnetohydrodynamic nanofluid flow with nonlinear radiation and viscous dissipation [Contribution 5], and examine heat transfer in porous fins with internal heat generation [Contribution 8] and entropy generation in rotating nanofluid flows [Contribution 15]. The performance limits of liquid metal microchannel heat sinks under high temperatures are also evaluated numerically [Contribution 17]. Together, these works provide deeper insights into convective heat transfer, flow control, and the impact of multi-physics coupling in single-phase systems.

Gao et al. [Contribution 2] reports that a variety of needle-motion profiles are used in diesel injectors. However, it is unclear as to what the underlying mechanism that determines the needle-motion profiles is, and how the needle-motion profiles affect the spray dynamics. Examining how the spray dynamics will change if only altering the needle valve opening speed or closing speed while all other parameters are kept the same has been of significant interest. The different needle-motion profiles are obtained using a piezo nozzle (Nozzle #P) and a solenoid nozzle (Nozzle #S), which have identical nozzle geometry. By utilizing the X-ray imaging technique, it is observed that the average needle valve speed of Nozzle #P is 51% higher at the opening stage but 17% lower at the closing stage than Nozzle #S. When the needle valve lift is low (approximately 200 μm), the needle valve opening speed has a crucial effect on spray dynamics. The faster needle valve opening of Nozzle #P results in a 42% larger spray spreading angle and 34% lower spray velocity at the downstream field. The spray dynamics may be controllable by properly designing the needle-motion profiles in the scenarios of the low needle lifts. However, when the needle valve is sufficiently open (approximately over 200 μm), almost identical spray characteristics are observed, regardless of the needle-motion profiles.

Bafakeeh et al. [Contribution 5] numerically studies the radiated flow of magnetized viscous fluid that is subject to the viscous dissipation phenomenon. The radiative phenomenon is addressed with nonlinear relations. Further, analysis is performed by using the slip effects and convective thermal flow constraints. The transformed problem is numerically evaluated using the Keller Box method. The physical parameter effects—such as the magnetic parameter for the velocity profile, the Prandtl number, the Brownian motion parameter, and the Biot number for the energy profile and Lewis number—and the thermophoresis parameter for the concentration profile are discussed. The obtained results suggest applications in enhancing the heat transfer phenomenon, thermal system, energy generation, heat transmission devices, power generation, chemical reactions, etc.

Sowmya et al. [Contribution 8] uses a variety of methodologies to explore heat transport enhancement; using the fin approach to inspect heat transfer characteristics is one such effective method. In a broad range of industrial applications, including heat exchangers and microchannel heat sinks, fins are often employed to improve heat transfer. Encouraged by this feature, the present research is concerned with the temperature distribution caused by convective and radiative mechanisms in an internal heat-generating porous longitudinal dovetail fin (DF). The Darcy formulation is considered for analyzing the velocity of the fluid passing through the fin, and the Rosseland approximation determines the radiation heat flux. The heat transfer problem of an inverted trapezoidal (dovetail) fin is governed by a second-order ordinary differential equation (ODE); to simplify it to a dimensionless form, nondimensional terms are utilized. The generated ODE is numerically solved by using the spectral collocation method (SCM) via a local linearization approach. The effect of different physical attributes on the dimensionless thermal field and heat flux is graphically illustrated. As a result, the temperature in the dovetail fin transmits in a decreasing manner for growing values of the porosity parameter. For elevated values of heat generation and the radiation-conduction parameter, the thermal profile of the fin displays an increase, whereas an increment in the convection–conduction parameter downsizes the thermal dispersal. It is found that the SCM technique is very effective and more conveniently handles the nonlinear heat transfer equation. Furthermore, the temperature field results from the SCM-based solution are in very close accordance with the outcomes published in the literature.

Ali et al. [Contribution 15] discuss entropy generation analysis for a peristaltic flow in a rotating medium with generalized complaint walls. The goal of the current analysis is to understand the fluid flow phenomena that are particular to micro devices. Nano materials with a size of less than 100 nm have applications in micro heat exchangers to cool electronic circuits, blood analyzers, biological cell separations, etc. For this study, they consider the effects of radiation, viscous dissipation, and heat flux on the flow of nanomaterial inside a cylindrical micro-channel. To investigate the slip effects on the flow, they use the second order slip condition for axial velocity, the first order slip condition for secondary velocity, and the thermal slip conditions. The flow is governed by partial differential equations (PDEs), which are turned into a system of coupled ordinary differential equations (ODEs) that are highly non-linear and numerically solved using the NDSolve command in Mathematica. The impacts of different involved parameters on the flow field are investigated with the aid of graphical illustrations. Entropy generation and the Bejan number are given special attention, and it is found that they decrease as the Hartman number, rotation, and radiation parameters increase.

Developments in applications such as rocket nozzles, miniature nuclear reactors, and solar thermal generation pose high-density heat dissipation challenges. In these applications, a large amount of heat must be removed in a limited space under a high temperature. Wu et al. [Contribution 17] propose liquid metal-based microchannel heat sinks to handle this kind of cooling problem. Using a numerical method, the flow and heat transfer performances of liquid metal-based heat sinks with different working fluid types, diverse microchannel cross-section shapes and various inlet velocities are studied. By solving the 3D steady and conjugate heat transfer model, they find that among all the investigated cases, lithium and circle are the most appropriate choices for the working fluid and microchannel cross-section shape, respectively. Moreover, the inlet velocity had a great influence on the flow and heat-transfer performances. From 1 m/s to 9 m/s, the pressure drop increases as much as 65 times, and the heat-transfer coefficient is enhanced by about 74.35%.

## 3. Mechanisms of Multi-Phase Flow

This category delves into the core interfacial and phase-change physics that are dominant at micro-scales. Research topics include the significant enhancement of evaporation via the Marangoni effect [Contribution 1], unique condensation frosting characteristics at plate edges [Contribution 3], mass transfer mechanisms for gas replenishment on hydrophobic surfaces [Contribution 4], particle deposition interactions near film cooling holes [Contribution 13], the non-uniform cooling efficiency of sintered wire mesh [Contribution 14], and the dynamic behavior of cavitation bubbles under actuation [Contribution 16]. These studies reveal the critical role of interfacial forces and phase interactions in multiphase systems.

Liu et al. [Contribution 1] built a coupled thermal mass model of droplet evaporation and test the accuracy of the numerical model through theoretical results to study the droplet heat and mass transfer law in the droplet evaporation process. In order to study the influence of the Marangoni effect on the droplet evaporation process and the effects of different initial droplet radii and an ambient temperature on the temperature and flow, fields within the droplet are compared. From this result, it can be seen that the droplet volume is 20 μL, and the maximum flow velocity in the droplet is 0.34 mm/s, without taking the Marangoni effect into account. When the Marangoni effect is considered, the maximum flow velocity increases by almost 100 times. The Marangoni effect can cause the convection in the droplet to change direction, and the formation of the Marangoni flow may affect the temperature distribution within the droplet, thereby increasing the evaporation efficiency by 2.5%. The evaporation process will increase the velocity of the air that is close to the surface of the liquid, but the increase in air velocity close to the liquid surface is not sufficient to reinforce evaporation. There is a non-linear relationship between an increasing ambient temperature and increasing evaporation efficiency. For every 5 °C increase in the ambient temperature, the maximum increase in the rate of evaporation is approximately 22.7%.

Zhang et al. [Contribution 3] conduct an experimental study on the localized condensation frosting characteristics in the edge region of a cold plate. The edge effects on the water droplet condensation (WDC), water droplet frozen (WDF), and frost layer growth characteristics are quantitatively investigated. The results show that the number of droplets coalescing in the edge-affected regions is around 50% greater than in the unaffected regions. At the end of the WDC stages, the area-average equivalent contact diameter and coverage area ratio of water droplets in the edge-affected regions are 2.69 times and 11.6% greater than those in the unaffected regions under natural convection, and the corresponding values are 2.24 times and 9.9%, respectively, under forced convection. Compared with the unaffected regions, the WDF stage duration in the edge-affected regions decrease by 63.6% and 95.3% under natural and forced convection, respectively. Additionally, plate-type and feather-type frost crystals are, respectively, observed in natural and forced convection. The results of this study can help us to better understand the condensation frosting mechanism on a cold plate, which in turn provides guidelines for optimizing the design of heat exchanger structures and system control strategies facing frosting problems.

The underwater nonwetted state on a superhydrophobic surface is hardly maintained in flowing water because the entrapped gas dissolves into the water or is carried off by the flow. Therefore, a source gas is necessary to maintain a superhydrophobic state for its applications under realistic conditions. As detailed in this paper, based on the gas entrapped on a hydrophobic structured surface, the gas regeneration is experimentally achieved to replenish the losses of gas that are carried off by the flow and reduced through dissolution. Wang et al. [Contribution 4] investigate the mechanism of mass transfer at the liquid–gas interface by simulation. The results indicate that the water molecules at a liquid–gas interface escape to the entrapped gas when the water content does not reach saturation. This phenomenon could be due to the evaporation at the liquid–gas interface. With the increasing water content in the entrapped gas, the evaporation rate at the liquid–gas interface descends gradually. Under the action of flowing, the substances containing high concentrations of water molecule are washed away at the liquid–gas interface. Therefore, the low concentration of the water molecule at the liquid–gas interface is created. As a result, the equilibrium of water and gas at the liquid-gad interface is broken, and the evaporation continued to replenish the lost gas. Overall, the presented results in this study could be considered to be a promising candidate for replenishing the lost gas in hydrophobic structured surfaces by mass transfer at the liquid–gas interface.

Peng et al. [Contributions 13, 14] present a combined experimental and numerical investigation on the particle deposition in the vicinity of multiple film cooling holes to reveal the effect of interactions between cooling outflows on particle deposition. They experimentally investigate the transpiration cooling characteristics of a porous material: sintered wire mesh. Three samples with different porosities in a plain weave structure are tested with various blowing ratios in an open-loop wind tunnel with a heated mainstream flow. The temperature on the surface of the porous material is measured by an infrared camera to obtain the cooling efficiency. The measurements reveal nonuniform distributions of the surface temperature and cooling efficiency in both the flow direction and the transverse direction. The averaged cooling efficiency on the surface first decreases and then increases with the blowing ratio, but it increases and then decreases with the porosity of the material. The internal cooling by forced convection and its combination with the external film cooling from the transpiration cooling are considered to be attributed to those two cooling characteristics, respectively. Finally, they propose a modified blowing ratio to collapse the minima of the blowing ratio for all tested samples, providing a universal transition for the decreasing and increasing branches for all tested samples in the relationship between the averaged cooling efficiency and the blowing ratio.

Shang et al. [Contribution 16] experimentally and numerically study the dynamics of cavitation bubbles in a nozzle-shaped microfluidic channel with PZT (lead zirconate titanate) actuations. It is found that a cloud of bubbles can be generated near the center of the microfluidic channel when the actuation voltage is larger than the threshold of 1 kHz. After being generated, the bubbles under actuations oscillate radially with violent expansion and compression, and they simultaneously translate upstream towards the opening of the nozzle. Along with radial oscillation and translation, the bubbles undergo frequent and drastic coalescence and breakup, leading to vigorous churning of the surrounding liquids. The pressure variation and distribution in the microchannel are calculated by numerical simulation in Ansys Fluent, and results show that there is a low-pressure zone inside the microfluidic channel within each cycle of the actuation period, which is responsible for the observed bubble generation in the experiments. The method of bubble generation in this study is novel and can be applied for the enhancement of heat and mass transfer in microfluidic operations.

## 4. Applications for Single- and Multi-Phase Flows

Focused on design optimization and performance analysis for engineering systems, this segment includes a numerical comparison of fin shapes for microchannel evaporator efficiency [Contribution 6], a parametric study of supercritical CO_2_ heat transfer in a spiral channel gas cooler [Contribution 9], the investigation of a reversed heat flux phenomenon in wire-wrapped nuclear fuel rods [Contribution 11], and experimental–analytical analysis of transient refrigerant behavior in microchannels [Contribution 12]. These works aim to solve practical thermal management challenges. This area addresses the performance characterization and operational limits of materials and systems involving phase change. One study measures and models the thermal conductivity of flexible composite phase-change materials [Contribution 7], while the other analytically determines the maximum heat load and temperature response of flat micro heat pipes with different working fluids [Contribution 10]. The findings offer vital data and guidelines for the design of reliable phase-change-based thermal systems.

Nguyen et al. [Contribution 6] numerically study the fin shape of a microchannel evaporator in a CO_2_ air conditioning system. The study is performed at the inlet evaporative temperature of 10 °C and the vapor quality of 0.61. Two types of fin shapes are examined: straight fins and V-fins. The numerical results are verified by the experimental data. For the system under consideration and for the same heat-transfer area and the heat-transfer coefficient for the air side in the microchannel evaporator, the effect of the fin shape on the heat transfer is not different; however, the solution time and the physical memory for the straight fins are 1.3 and 1.45 times longer than those of the V-fins, respectively. Therefore, the V-fin shape should be used for numerical simulation to compare it with the straight fin shape. In this study, the evaporation of the refrigerant in the microchannel evaporator takes place in four passes. The normal heat flux from the air through the fins and tubes is almost reached at 1550 W/m^2^, at the evaporative temperature of 10 °C. The obtained results from the experimental data are in good agreement with those obtained from the numerical results, with a deviation of less than 10%.

Jiang et al. [Contribution 9] study a heat exchanger with a spiral channel. ANSYS CFX software is used to analyze the flow and heat-transfer characteristics of the heat exchanger (single-plate model). The influences of the cooling pressure of CO_2_, the mass flux of CO_2_, the mass flux of water, and the channel radius of CO_2_ are discussed. In this paper, the results show that the cooling pressure of CO_2_, the mass flux of CO_2_, and the channel radius of CO_2_ all have a large influence on the local heat transfer coefficient: with an increase in the cooling pressure of CO_2_, the peak value of the heat transfer coefficient of CO_2_ decreases and the average heat transfer coefficient decreases; with an increase in the mass flux of CO_2_, the peak value of the heat transfer coefficient of CO_2_ increases and the average heat transfer coefficient increases; and with a decrease in the channel radius of CO_2_, the peak value of the heat transfer coefficient of CO_2_ increases. The water mass flux only has a small effect on the heat transfer, and the lower cooling pressure of CO_2_ corresponds to a higher peak heat transfer coefficient, which can reach 27.5 kW m^−2^ K^−1^ at 9 MPa. A gas cooler is one of the important parts of a carbon dioxide (CO_2_) heat pump water heater, and it must meet the needs of both pressurization and heat transfer. It is important to study gas coolers. 

Tan et al. [Contribution 11] discover the existence of reversed heat flux from coolant to wrapped wire, which is contrary to our usual understanding. This phenomenon has not been reported in previous CFD calculations. Hence, a solid heat-conduction model is proposed to prove this phenomenon and analyze the heat-transfer process. The simulation results show that the wrapping wire embedding depth, the shape of the calculation domain, and the physical properties of all components have great influence on the magnitude of the reversed heat flux. The present findings will have a strong influence on the temperature field and maximum value of the fuel rod, as well as profound reference value for future flow calculation, especially in grid generation and treatment of the junction between the winding wire and fuel rod.

Mihai et al. [Contribution 12] conduct an analysis of the R134a (tetrafluoroetane) coolant’s non-stationary behavior in rectangular microchannels with the help of a newly proposed miniature refrigerating machine of their own design and construction. The experimental device incorporates a condenser, a lamination tube and a vaporizer on the same plate, all of which integrate rectangular microchannels. The size of the rectangular microchannels is determined by laser profilometry. R-134a coolant vapors are pressurized using a small ASPEN rotary compressor. By using the variable soft spheres (VSS) model, the mean free path, Knudsen and Reynolds numbers, and the dimensionless velocity profile can be assessed analytically. In order to determine the average dimensionless temperature drop in the vaporizer’s rectangular microchannels, in a non-stationary regime, an analytical solution for incompressible flow with slip at the walls, fully developed flow, and a laminar regime is used, with the aid of an integral transform approach. In the experimental study, the transitional distribution of temperature is tracked while modifying the R134a flow through the rectangular microchannels. Coolant flow is then maintained at a constant, while the amount of heat absorbed by the vaporizer was varied using multiple electric resistors. A comparative analysis of the analytical and experimental values is conducted.

Phase-change materials (PCMs) are widely used in energy storage and thermal management due to the large amount of latent heat in the phase-change process. As one of the most significant thermophysical properties of PCMs, the thermal conductivity has been extensively studied. Great attention has been paid to improving the thermal conductivities of PCMs; however, the studies on the thermal conductivities of flexible PCMs are relatively inadequate. Feng et al. [Contribution 7] use polyethylene glycol 1500 (PEG1500) as the base PCM, and expanded graphite (EG) and styrene–butadiene–styrene (SBS) are added to improve the thermal conductivity and flexibility of pure PCMs, respectively. A steady-state experimental test rig is built and verified with the measurement of the thermal conductivity of stainless steel and deionized water, and then the thermal conductivities of PCMs at different phases and qualitative temperatures are measured extensively. Compared to the PEG1500 with 5 wt.% EG, the addition of SBS sharply reduces the thermal conductivity, which is only 0.362 W/(m·K) at 12.5 °C when the addition ratio is 50%. This is approximately a 69% reduction compared with the composite PCMs without SBS. Furthermore, the theoretical thermal conductivities of the composite PCMs are calculated with six theoretical models of multiphase systems. The majority of the models provide a good prediction for the thermal conductivities of composite PCM with high SBS concentration, while the average deviation of the Agari–Uno model is only 20.5% with a different SBS concentration and, relatively, it agrees well with the experimental results.

Mihai et al. [Contribution 10] report a series of considerations for the occurrence of capillary boundaries in flat micro heat pipes (flat MHPs) and the conditions required for their stable operation in relation to the working circumstances and to the type of liquids inside the pipes. The particularities of heat transfer in a flat MHP are analyzed for situations with either excessive or deficient working liquid. Depending on the physical properties of the working liquids (acetone, methanol, and distilled water), the maximum rate of heat flow that can be applied to a flat MHP is determined analytically. The calculus is made with the assumption that constant vaporization of the liquid is ensured in the flat MHP’s evaporator, with no overheating. The analytical models considered here allow for the evaluation of the liquid film thickness and the mass flow corresponding to the vaporization region. The temperature difference between the inner and outer walls of a flat MHP is found in the case of a transient regime and a variable thermal flow is applied in the evaporation region. The interior of the flat MHPs is modeled in MATLAB, using an FTCS (Forward Time Central Space) method, which is a finite difference method that is used to numerically solve the heat equation.

To conclude, we would like to acknowledge all the authors for their contributions to the success of this Special Issue on Heat and Mass Transfer in Microchannels, as well as the reviewers whose feedback helped to improve the quality of the published papers.

## Data Availability

The raw data supporting the conclusions of this article will be made available by the authors on request.
